# How safe is diathermy in patients with cochlear implants?

**DOI:** 10.1308/003588412X13373405386538

**Published:** 2012-05

**Authors:** SJ Frampton, H Ismail-Koch, TE Mitchell

**Affiliations:** ^1^The South of England Cochlear Implant Centre,UK; ^2^University Hospital Southampton NHS Foundation Trust,UK

**Keywords:** Cochlear implants, Electrocoagulation, Electrosurgery, Equipment failure

## Abstract

**INTRODUCTION:**

Cochlear implants are surgically inserted electrical devices that enable severely or profoundly deaf individuals to interpret sounds from their environment and communicate more effectively. As a result of their electrical nature, they are susceptible to electromagnetic interference and can be damaged by excessive electrical energy. Surgical diathermy is one source of such potentially damaging energy. The British Cochlear Implant Group guidelines advise that monopolar diathermy should not be used in the head and neck region in patients with cochlear implants and that bipolar diathermy should not be used within 2cm of the implant (http://www.bcig.org.uk/site/public/current/safety.htm).

**METHODS:**

A questionnaire was provided to 36 surgeons working in different specialties in the head and neck region, inquiring as to their knowledge of the safety considerations when using diathermy in cochlear implant patients. Thirty-five surgeons provided responses.

**RESULTS:**

Overall, 77% of the respondents were unaware of the existence of published guidelines. Even when given an option to seek advice, 11% erroneously felt it was safe to use monopolar diathermy above the clavicles with a cochlear implant in situ and 49% felt that there was no restriction on the use of bipolar diathermy.

**CONCLUSIONS:**

There is a significant deficit in the knowledge of safe operating practice in the rapidly expanding population of patients with cochlear implants which threatens patient safety. Through this publication we aim to increase awareness of these guidelines among members of the surgical community and this paper is intended to act as a point of reference to link through to the published safety guidelines.

Cochlear implants are surgically inserted electrical devices designed to stimulate the auditory nerve and bypass the defective hair cells of the cochlea. The implant consists of a receiver-stimulator package that is secured under the periosteum of the cranium, above and behind the ear, connected to an array of electrodes (Fig 1). This electrode array is surgically inserted into the cochlea via a route drilled through the mastoid bone. Sounds from the patient’s environment are received by a microphone worn either behind the ear or elsewhere on the body and transformed into a digital electrical signal by a speech processor in the same unit.

**Figure 1:**
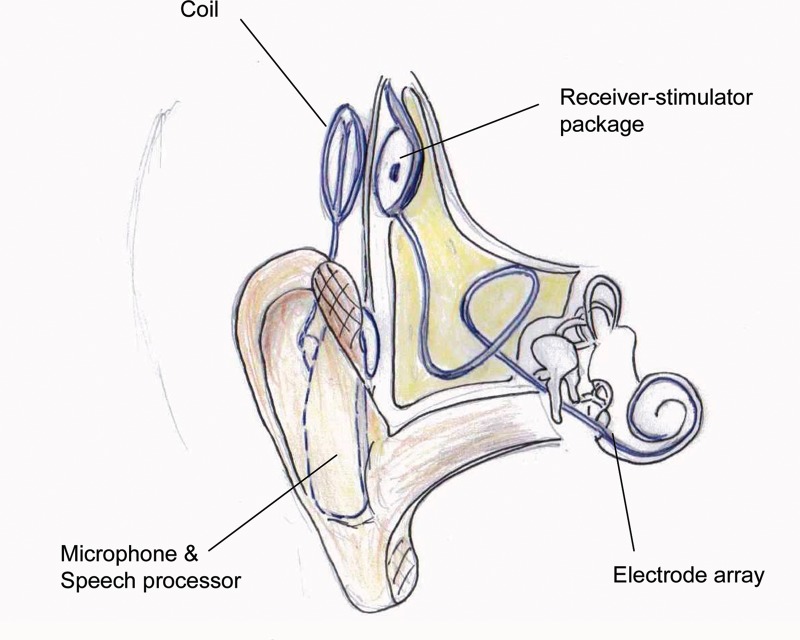
Schematic diagram of cochlear implant in situ

The digital signal is transferred to the implanted receiver-stimulator package via a coil that sits on the surface of the skin over the package and that is connected by a wire to the speech processor. The coil is held in place over the implanted receiver-stimulator by a magnet and a radio frequency signal is transmitted across the skin. The speech processing software mimics the tonotopic organisation of the human cochlea so that different frequencies of environmental sound are relayed to the brain by stimulation of the spiral ganglion cells at different points in the cochlea.

Surgical diathermy employs high-frequency alternating current, which produces heat when applied to tissues, to cut, desiccate or coagulate them. Bipolar diathermy allows current to flow only between the opposing tines of the instrument while the current in monopolar diathermy passes from the handpiece, through the body, to the return electrode plate, which is applied to skin distant from the surgical field. The presence of electrical current in the vicinity of a cochlear implant can be damaging and may necessitate replacement of the damaged implant with the associated risks of further surgery and significant additional expense.

The British Cochlear Implant Group (BCIG) safety guidelines advise that monopolar diathermy should not be used in the head and neck region in patients with cochlear implants. Bipolar diathermy can be used safely but should not be used within 2cm of the implant.

The aim of this study was to assess the awareness of these guidelines among surgeons operating in the head and neck region, and to raise their profile in the surgical community in order to reduce the risk of iatrogenic damage to these expensive and sensitive devices.

## Methods

Surgeons operating in the head and neck region in adults and children in one teaching hospital and one district general hospital were asked to complete a short questionnaire using only their existing knowledge. Surgeons trained in the insertion of cochlear implants were not included in the study.

## Results

There was a 97% response rate (35/36) from individuals who were approached. Surgeons from the departments of otolaryngology, oral and maxillofacial surgery, dermatology, plastic surgery and paediatric surgery contributed to the survey. Sixteen surgeons were consultant grade, two were associate specialists and seventeen were middle grades (specialist registrars, staff grade or specialist doctors).

Only 8/35 surgeons (23%) operating in the head and neck region were aware of the existence of any guidelines on the use of diathermy in patients with cochlear implants.

Four surgeons (11%) felt erroneously that monopolar diathermy was safe to use in the head and neck region in patients with cochlear implants while eleven (31%) felt correctly that it was unsafe to use and the remainder chose to seek advice.

Only two of the surgeons questioned (6%) correctly that distance from the implant was important when using bipolar diathermy but none knew the recommended distance. Seventeen (49%) stated that they would use bipolar diathermy without restriction in patients with cochlear implants and three (9%) stated that they would use a lower power setting. The remainder stated that they would seek advice.

Of the nine surgeons who had knowingly operated on patients with cochlear implants, five (56%) were unaware of published guidance on the use of diathermy. Of the same nine surgeons, one (11%) stated that he or she would have been happy to use monopolar diathermy above the clavicles in these patients while five (56%) knew that monopolar diathermy should not be used. The remainder stated that they would have sought advice. Two (22%) of these nine surgeons correctly felt that bipolar was safe to use a specified distance from the implant, one (11%) felt that it could be used anywhere at a lower power setting, and two (22%) would not restrict their bipolar use in any way in cochlear implant patients. The remaining four (44%) would have sought advice.

## Discussion

The first cochlear implant was attempted in a living human in 1957.^1^ The crude single channel device has undergone significant technological development over the past 50 years to become an efficient and reliable^2^ device produced by several manufacturers. In 2011 there were approximately 11,000 cochlear implant users in the UK alone^3^ and at the end of the previous year there were 219,000 users worldwide.^4^

Technology appraisal guidance from the National Institute for Health and Clinical Excellence (NICE) currently exists for cochlear implantation in the UK National Health Service (NHS).^5^ With careful patient selection, most of the individuals implanted have improved sound perception compared with their prior use of hearing aids. Although the percepts of sound are very different from those created naturally in a normal hearing ear, with learning they can be interpreted effectively so that over 70% of implanted individuals can understand speech using the telephone.^6^ The remainder gain considerable benefit from the use of sound to augment lipreading comprehension and from the awareness of environmental sounds. Implantation in young children, typically around 12 months of age, can allow normal speech and language development, which would otherwise be impossible. This improves their educational potential dramatically and allows many children to enter mainstream education.^7^

Approximately 370 children are born in England and Wales each year with severe-profound deafness and by the age of 3 years roughly 1 in 1,000 children experience this degree of hearing impairment.^5^ The newborn hearing screening programme, introduced in England in 2006, has facilitated earlier identification of newborn children requiring more detailed audiological assessment and early implantation where appropriate. In 2010, 500 children in the UK with severe-profound hearing loss received cochlear implants.^3^

Of the adult population in the UK, 613,000 have severe or profound hearing loss including 3% of the population aged over 50 and 8% of those over 70.^5^ In 2010, however, only 500 adult patients in the UK underwent cochlear implantation.^3^ It is likely that this number will increase significantly in the future.

The first cochlear implant in the UK was performed in the 1980s.^8^ Currently, NHS funding is available for unilateral implantation in adults and bilateral implantation in children who meet NICE audiological criteria. The current cost of assessment, implantation, and the first year of tuning and support is approximately £30,000 for unilateral adult implantation and £45,000 for bilateral implantation in children.

Unfortunately, the implanted portion of these devices occasionally fails. Minor electronic problems can sometimes be ‘tuned out’ or the programme in the speech processor reset but problems may be progressive, necessitating removal of the implant and the insertion of a replacement. This is not only financially punitive but also exposes the patient to risks of repeat surgery including facial nerve injury, taste disturbance, vertigo, tinnitus and wound infection.

Cochlear implants, being complex electrical devices, using small currents, are particularly sensitive to electrical interference. Even the static charge released from some articles of clothing can be sufficient to corrupt the speech processor electronics or damage implant components^9^ and interference can be experienced from electrical devices such as mobile phones or overhead power lines.^10^ It is therefore not surprising that the electrical currents used in surgical diathermy can permanently damage the implanted electronics, causing device failure. Furthermore, such currents may produce irreversible damage to the cochlear tissues, negating any potential benefit gained from reimplantation.^9^

The use of monopolar diathermy below the clavicles is not thought to pose a threat to the implant system, as shown by electrical and behavioural testing in cochlear implant patients who have undergone coronary artery bypass grafting using monopolar diathermy.^11^ Above the clavicles, monopolar diathermy has been shown to be damaging to the cochlear implant circuitry in some^12^ but not all cadaveric studies.^13^ However, even in those cadaveric studies where no permanent damage to the circuitry could be identified, it is not known whether damage to the cochlear tissues with a resultant change in the percept of hearing would have occurred.^13^

In collaboration with the cochlear implant manufacturers, the BCIG has produced guidance for patients and healthcare professionals on lifestyle activities and medical interventions that can affect implant recipients.^10^ Their advice is that monopolar diathermy should not be used in the head and neck region on patients with cochlear implants and that bipolar diathermy should not be used within 2cm of the implant.

## Conclusions

While responsibility for caring for an implant is shared between the patient and subsequent caregivers, many patients do not understand the concept of diathermy and do not feel able to advise medical professionals regarding surgical technique. Many implant recipients do not carry their cochlear implant identification cards, which could otherwise act as a reference point for medical staff. This study indicates that there is insufficient awareness among surgeons of the published safety guidelines and of safe practice using diathermy in the head and neck region in these patients.

The unsafe use of diathermy risks irreversible damage to the implant, which may necessitate costly reimplantation with the inherent risk of permanent and significant surgical complications for the patient. Furthermore, currents transmitted to the cochlea may theoretically permanently damage inner-ear neural tissue, negating any benefit of reimplantation of a new device. All surgeons operating in the head and neck region should be aware of the existence of these guidelines and consult them, or seek advice, when presented with a patient fitted with a cochlear implant.
